# Targeted Sequencing of 10,198 Samples Confirms Abnormalities in Neuronal Activity and Implicates Voltage-Gated Sodium Channels in Schizophrenia Pathogenesis

**DOI:** 10.1016/j.biopsych.2018.08.022

**Published:** 2019-04-01

**Authors:** Elliott Rees, Noa Carrera, Joanne Morgan, Kirsty Hambridge, Valentina Escott-Price, Andrew J. Pocklington, Alexander L. Richards, Antonio F. Pardiñas, GROUP Investigators, Behrooz Z. Alizadeh, Therese van Amelsvoort, Agna A. Bartels-Velthuis, Nico J. van Beveren, Richard Bruggeman, Wiepke Cahn, Lieuwe de Haan, Philippe Delespaul, Carin J. Meijer, Inez Myin-Germeys, Rene S. Kahn, Frederike Schirmbeck, Claudia J.P. Simons, Neeltje E. van Haren, Jim van Os, Ruud van Winkel, Jurjen J. Luykx, Colm McDonald, Gary Donohoe, Derek W. Morris, Elaine Kenny, Eric Kelleher, Michael Gill, Aiden Corvin, George Kirov, James T.R. Walters, Peter Holmans, Michael J. Owen, Michael C. O’Donovan

**Affiliations:** a MRC Centre for Neuropsychiatric Genetics and Genomics, Division of Psychological Medicine and Clinical Neurosciences, School of Medicine, Cardiff University, Cardiff, United Kingdom; b Centre for Neuroimaging and Cognitive Genomics, National University of Ireland Galway, Galway, Ireland; c Department of Psychiatry and Trinity Translational Medicine Institute, Trinity College Dublin, Dublin, Ireland

**Keywords:** ARC, Genetics, NMDAR, Schizophrenia, Sequencing, Voltage-gated sodium channels

## Abstract

**Background:**

Sequencing studies have pointed to the involvement in schizophrenia of rare coding variants in neuronally expressed genes, including activity-regulated cytoskeleton-associated protein (ARC) and *N*-methyl-D-aspartate receptor (NMDAR) complexes; however, larger samples are required to reveal novel genes and specific biological mechanisms.

**Methods:**

We sequenced 187 genes, selected for prior evidence of association with schizophrenia, in a new dataset of 5207 cases and 4991 controls. Included among these genes were members of ARC and NMDAR postsynaptic protein complexes, as well as voltage-gated sodium and calcium channels. We performed a rare variant meta-analysis with published sequencing data for a total of 11,319 cases, 15,854 controls, and 1136 trios.

**Results:**

While no individual gene was significantly associated with schizophrenia after genome-wide correction for multiple testing, we strengthen the evidence that rare exonic variants in the ARC (*p =* 4.0 × 10^–4^) and NMDAR (*p =* 1.7 × 10^–5^) synaptic complexes are risk factors for schizophrenia. In addition, we found that loss-of-function variants and missense variants at paralog-conserved sites were enriched in voltage-gated sodium channels, particularly the alpha subunits (*p =* 8.6 × 10^–4^).

**Conclusions:**

In one of the largest sequencing studies of schizophrenia to date, we provide novel evidence that multiple voltage-gated sodium channels are involved in schizophrenia pathogenesis and confirm the involvement of ARC and NMDAR postsynaptic complexes.

Schizophrenia is a highly heritable polygenic disorder (1). Collectively, common variants contribute up to half of the genetic variance in schizophrenia liability 2, 3, and 145 distinct loci have currently been associated with the disorder at genome-wide levels of significance in the most recent genome-wide association study (4). Schizophrenia risk is also conferred by rare mutations, including copy number variants (CNVs) 5, 6 and rare coding variants (RCVs) 7, 8, each of which sometimes occur as de novo mutations 9, 10.

Studies of RCVs have the potential to inform schizophrenia pathogenesis because they can pinpoint specific functional variants in individual genes. However, only two genes, *SETD1A* (11) and *RBM12* (12), have been strongly implicated. A major limiting factor, as for studies of common variants, is that for complex disorders, large samples are required to obtain robust results in case-control studies (13). To date, the largest published sequencing studies of schizophrenia have involved around 5000 cases, 9000 controls, and 1000 parent-proband trios 7, 11, almost an order of magnitude smaller than recently published schizophrenia single nucleotide polymorphism genotyping studies of common risk variants [e.g., 40,675 cases and 64,643 controls (4)]. Nevertheless, exome sequencing studies have provided important clues to the pathophysiology of schizophrenia. For example, proband-parent trio-based studies have shown de novo RCVs to be significantly enriched among glutamatergic postsynaptic proteins, in particular, the activity-regulated cytoskeleton-associated protein (ARC) and *N*-methyl-D-aspartate receptor (NMDAR) complexes (9). These synaptic gene sets, first associated with schizophrenia through studies of de novo CNVs (10), have also shown evidence for association in independent case-control CNV (14) and sequencing 7, 8 datasets. More recently, in an extension of the Swedish sample used by Purcell *et al.* (8), the authors documented an elevated exome-wide burden of ultra-rare, protein disruptive variants, which was concentrated among 3388 neuron-specific genes, particularly those that are expressed at synapses, including the ARC and NMDAR complexes (7). Additionally, the enrichment of RCVs in schizophrenia has been shown to be concentrated among 3488 genes that are depleted for loss-of-function (LoF) mutation in large population cohorts 15, 16.

In the current study, we performed targeted sequencing of 187 genes, selected for prior evidence for association with schizophrenia (Table S1 in Supplement 2), in 5207 cases and 4991 controls, none of which have contributed to previous schizophrenia sequencing studies. Among these targeted genes, we had complete membership of four gene sets: ARC and NMDAR postsynaptic protein complexes, which have been strongly implicated in multiple previous studies 9, 10, and voltage-gated sodium (17) and calcium (8) channels, which have inconclusive evidence for association with schizophrenia in previous rare variant studies 7, 17. The remainder of the genes targeted for sequencing were selected on the basis of supportive evidence from at least two sources (see Methods and Materials). Our primary aims were to 1) test for enrichment of RCVs in all 187 targeted genes, 2) test for enrichment of RCVs in four candidate gene sets previously implicated in schizophrenia, and 3) identify individual genes significantly enriched for RCVs.

Most recent studies of RCVs in schizophrenia have focused on LoF variants. However, it is clear that missense variants also contribute to schizophrenia risk 7, 9, but in contrast to LoF variants, in silico methods cannot distinguish at high sensitivity and specificity between missense variants that alter the function of the encoded protein and those that are benign. Recently, it has been shown that restricting analyses to missense variants affecting amino acids that are conserved within paralogous gene families improves power for identifying pathogenic variants (18). Given that two of our targeted gene sets consist of paralogous gene families (voltage-gated sodium and calcium channels), we exploited this approach in a secondary analysis of paralog-conserved missense variants (18).

Finally, to maximize power, we meta-analyzed the new sequencing data with independent, published schizophrenia case-control [Swedish (7) and UK10K (11) datasets] and trio exome-sequencing data (see Methods and Materials), yielding a combined analysis of RCVs in a total of 11,319 cases, 15,854 controls, and 1136 trios.

## Methods and Materials

### Ethics Statement

All research conducted as part of this study was consistent with UK regulatory and ethical guidelines. We gained national National Health Service research ethics committee approval for the CLOZUK (10/WSE02/15) and Cardiff COGS (07/WSE03/110) studies. The control samples were recruited as part of independent projects, all of which have equivalent ethical permissions and data sharing procedures in place.

### Sample Description

#### Targeted Sequence Sample

A total of 11,493 blood-derived DNA samples were selected for targeted sequencing (5724 cases and 5769 controls). None have been included in previous schizophrenia sequencing studies. The majority of sequenced cases were from the CLOZUK dataset (*n =* 4647), which has been described previously (19) and in Supplement 1. We sequenced additional cases from the United Kingdom (Cardiff COGS cohort; *n =* 521), Ireland (Dublin cohort; *n =* 335), and the Netherlands [GROUP cohort (20); *n =* 221]. We sequenced UK controls from the Wellcome Trust Case Control Consortium 2 consortium (1958 birth cohort, *n =* 2860; UK blood donors, *n* = 2463) 21, 22, 23. Additional controls were sequenced from the Dublin (*n =* 230) and GROUP (*n =* 216) cohorts (20). Sample descriptions are presented in Supplement 1.

#### Additional Datasets

We acquired publically available case-control exome sequencing data from the UK10K study (1352 cases and 4769 controls) (11) and a Swedish study (4867 cases and 6140 controls) (7). De novo mutations from 1136 published schizophrenia-proband parent trios were derived from published studies 9, 24, 25, 26, 27, 28, 29, 30, 31 (Table S8 in Supplement 1).

Power calculations for our final sample size are presented in Table S11 in Supplement 1.

### Gene Selection

We used Ion Torrent instruments (Thermo Fisher Scientific, Waltham, MA) to sequence the coding regions of genes belonging to the following gene sets: ARC (*n =* 28) (9), NMDAR (*n =* 61) (9), voltage-gated calcium channels (*n =* 26) (8), and voltage-gated sodium channels (*n =* 14) (17). We sequenced an additional 58 genes, selected for having two or more supportive lines of evidence for association with schizophrenia (full criteria for gene selection described in Supplement 1 and Table S1 in Supplement 2).

### Data Processing and Quality Control

The protocols used for targeted sequencing, data processing, and quality control are presented in Supplement 1. Briefly, raw sequence reads were independently processed for each Ion Torrent wave according to GATK best practice guidelines 32, 33. We excluded samples that were outliers from their sequencing wave’s mean for proportion of variants in the database of single nucleotide polymorphisms, number of alternative alleles, number of singletons, number of synonymous mutations, and number of nonsynonymous mutations. For 96% (5508 of 5724) of cases and 72% (4149 of 5769) of controls, we used available array data to identify and remove duplicate and first-degree relatives and samples with a genotype concordance <0.9. For samples not previously genotyped, we used Ion Torrent sequence data to exclude duplicate samples. Principal component analysis was used to identify and exclude cases and controls with non-European ancestry. After quality control, 5207 cases and 4991 controls from the targeted sequence sample, 4765 cases and 6107 controls from the Swedish sample, and 1347 cases and 4756 controls from the UK10K sample were retained for analysis. Variant annotation and quality control are described in Supplement 1.

### Statistics

Gene set and single-gene association statistics for case-control data were generated using the following Firth’s penalized-likelihood logistic regression model:

Logit(pr(case))∼Ntestvariants+baselinesynonymouscount+first10PCs+sex+IonTorrentsequencingwave(targetedanalysisonly)

The *p* values from the above models were compared with those generated in the same manner from 100,000 random permutations of case-control labels in our datasets. Enrichment for de novo mutations was tested using the statistical framework described in Samocha *et al.* (34), in which we compared the observed and expected number of de novo mutations using a Poisson test. A full description of our statistical approach for the above tests, and the case-control–de novo meta-analysis, can be found in Supplement 1.

### Approach to Hypothesis Testing and Multiple Testing

Here we outline our main enrichment tests and our approach for correcting for multiple testing (further details in Supplement 1). We first tested for the enrichment of RCVs in all 187 genes by performing six burden tests (LoF, nonsynonymous damaging, and nonsynonymous variant annotations under two allele frequency thresholds [<0.1% and singletons]). The derived *p* values were Bonferroni corrected for six tests. We then performed an exploratory analysis to further characterize any observed enrichments, by partitioning the targeted genes into those intolerant of LoF variants (pLi > 0.9) and those that are not (pLi ≤ 0.9). Because this later analysis was exploratory, no multiple testing correction was applied.

In our primary case-control gene set analysis, we had data for four sets; two synaptic sets (ARC and NMDAR) and two ion-channel sets (voltage-gated sodium channels and voltage-gated calcium channels). These were tested for enrichment of rare (<0.1% frequency) LoF variants, as this was the only class of mutation enriched among all 187 genes after correction for multiple testing (see Results). The *p* values derived from our new targeted sequencing sample were therefore Bonferroni corrected for four tests (four gene sets × one mutation class).

For meta-analysis, we note that the inclusion of ARC, NMDAR, and calcium-channel gene sets in the present study was predicated on previous associations from exome-wide de novo and case-control studies that are included in the present meta-analysis 8, 9. This ascertainment bias makes it impossible to generate meaningful and appropriately conservative study-wide multiple-testing corrections. Therefore, we consider those meta-analyses as representing an appraisal of the current sequencing evidence for those gene sets. The case-control meta-analysis of sodium channels does not include any previously reported data and therefore it does not suffer from such an ascertainment bias; accordingly, we calculate study-wide corrected *p* values as we did for the new sequencing data (four gene sets × one mutation class).

For the secondary analysis of LoF variants and missense variants at paralog-conserved sites, although this was only apropriate for the two ion-channel gene sets, aiming to be conservative, we Bonferroni corrected for eight potential tests (four gene sets × two mutation classes). To dissect the observed enrichment of LoF and missense variants at paralog-conserved sites in sodium channels (see Results), we partioned them into alpha and beta subunits. Aiming to favor caution in view of the novelty of the finding, we conservatively Bonferroni corrected the derived *p* values for 12 potential tests (two mutation classes tested against four gene sets plus the two subsets of sodium channel alpha and beta subunits).

For single-gene enrichment analysis of rare (< 0.1% frequency) LoF variants, we applied exome-wide criteria for multiple testing correction by Bonferroni correcting *p* values for 20,000 tests.

## Results

### Mutation Burden

In the targeted sequence sample, we performed six primary tests of mutation burden across all 187 targeted genes: LoF, nonsynonymous damaging, and nonsynonymous variants, each under two allele frequency thresholds (<0.1% and singletons). Correcting for six tests, we observed a significant (*p*_*corrected*_ < .05) excess of LoF mutations (< 0.1% frequency) in cases (Table 1), that had a mean excess of 0.013 LoF mutations/person across the 187 targeted genes (Table S2 in Supplement 2). Similar results were obtained by permutation analysis (*p =* .0013; *p*_*corrected*_ = .0078). There was no significant difference between cases and controls for any other class of variant (Table 1). As part of our quality control, we note no difference between cases and controls in the rate of synonymous mutation at the same frequency (<0.1%; odds ratio [OR], 1.02; 95% confidence interval [CI], 0.94–1.08; *p* = 1), suggesting that the enrichment of LoF mutations in cases is unlikely to be due to technical artifacts.

**Table 1:** Mutation Burden

Analysis	Genes Tested	Mutation Type	MAF <0.1%						Singletons					
			Targeted Sequencing Sample				Meta-analysis		Targeted Sequencing Sample				Meta-analysis	
			Mutations (Cases/Controls)	Rate (Cases/Controls)	*p*	OR (95% CI)	*p*	OR (95% CI)	Mutations (Cases/Controls)	Rate (Cases/Controls)	*p*	OR (95% CI)	*p*	OR (95% CI)
Primary	187 targeted genes	LoF	271/195	0.052/0.039	.0012^a^	1.36 (1.13–1.64)	.00035	1.22 (1.1–1.37)	94/66	0.018/0.013	.047	1.38 (1–1.9)	.0043	1.31 (1.09–1.57)
		NSD	5854/5425	1.12/1.09	.083	1.03 (1.00–1.07)	.93	1.00 (0.98–1.03)	1268/1223	0.24/0.25	.94	1.00 (0.92–1.08)	.98	1.00 (0.95–1.06)
		NS	9199/8625	1.77/1.73	.14	1.02 (0.99–1.05)	.85	1.00 (0.98–1.02)	1878/1845	0.36/0.37	.56	0.98 (0.92–1.05)	.38	0.98 (0.94–1.02)
Exploratory	106 LoF-intolerant genes	LoF	70/42	0.013/0.0084	.006	1.7 (1.16–2.53)	2.9 × 10^–6^	1.63 (1.33–2.0)	42/27	0.0081/0.0054	.1	1.49 (0.92–2.45)	.00045	1.67 (1.25–2.22)
	81 LoF-tolerant genes	LoF	201/153	0.039/0.031	.03	1.27 (1.03–1.57)	.21	1.09 (0.95–1.24)	52/39	0.01/0.0078	.23	1.29 (0.85–1.96)	.5	1.09 (0.85–1.39)

The targeted sequencing sample includes data from 5207 cases and 4991 controls. The meta-analysis includes data from 11,319 cases and 15,854 controls. Rates correspond to the average number of mutations per case/control. The *p* values are two-sided, and odds ratios (OR) and 95% confidence intervals (CIs) were generated from logistic regression models. In the targeted sequencing sample, *p* values that are in bold. The exploratory analysis was performed to determine whether the excess of rare (<0.1% frequency), loss-of-function (LoF) variants in all 187 genes is concentrated among genes known to be intolerant to this class of mutation.

MAF, minor allele frequency; NS, nonsynonymous; NSD, nonsynonymous damaging.

a Survived Bonferroni correction for multiple testing (six tests).

Meta-analysis with two previously published case-control exome sequencing datasets (Sweden and UK10K) strengthened the evidence for an increase in LoF variants (frequency <0.1%) in the set of 187 genes in cases (Table 1 and Table S3 in Supplement 2).

We partitioned the 187 genes into those intolerant of LoF variants [pLi scores > 0.9 in nonpsych-Exome Aggregation Consortium data (15)] and those that are not intolerant (pLi ≤ 0.9). Meta-analysis of the case-control data showed that association between schizophrenia and rare (frequency <0.1%) LoF variants was stronger in LoF-intolerant genes (Table 1; *Z*-test difference in effect size *p =* .0006).

### Gene Set Analysis

#### Primary Analysis of Voltage-Gated Sodium and Calcium Channels

We found nominally significant evidence for enrichment in cases for LoF variants (frequency <0.1%) in voltage-gated sodium channels (targeted sequencing sample: OR, 1.99; 95% CI, 1.11–3.71; *p =* .02; *p*_*corrected*_ = .08; case-control-de novo meta-analysis: *p =* .025; *p*_*corrected*_ = .1) (Table S4 in Supplement 2), but no evidence for association between schizophrenia and voltage-gated calcium channels (Table S4 in Supplement 2).

#### Secondary Analysis of Paralog-Conserved Ion-Channel Sites

**Figure 1:**
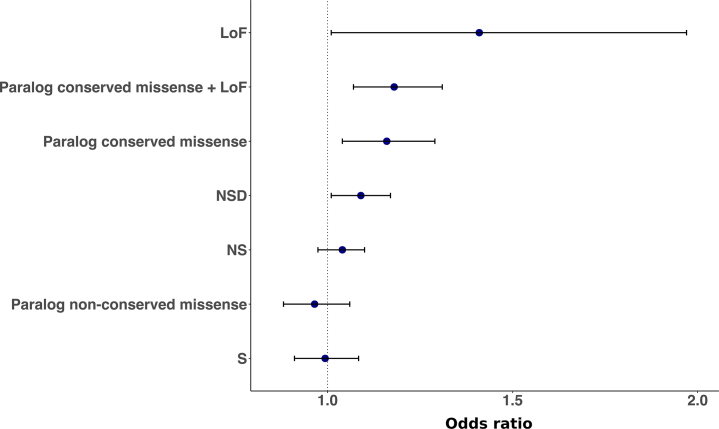
Case-control meta-analysis of rare (frequency <0.1%) variants in voltage-gated sodium channels. For comparison, we present results for variants outside those tested in our primary (loss-of-function [LoF]) and secondary (paralog-conserved missense and LoF) analyses, which include negative controls (synonymous [S] and paralog-nonconserved missense). NS, nonsynonymous; NSD, nonsynonymous damaging.

In the targeted sequence sample, we found a significant case excess of rare (frequency <0.1%) paralog-conserved missense and LoF variants in sodium channels (OR, 1.26; 95% CI, 1.08–1.47; *p =* .0035; empirical *p =* .0034; *p*_*corrected*_ = .027) but not calcium channels (Table S5 in Supplement 2). This enrichment was also supported in the full case-control meta-analysis (OR, 1.18; 95% CI, 1.07–1.31; *p =* .0014; *p*_*corrected*_ = .011) (Figure 1, Table S5 in Supplement 2).

The following exploratory analyses were conducted to test the robustness of the enrichment of paralog-conserved missense and LoF variants in sodium channels. We found evidence that the sodium-channel enrichment does not simply reflect a general increased burden for LoF variants and missense variants at paralog sites, as it is significantly greater than sets of genes of equivalent size sampled randomly from the non–sodium-channel component of our targeted gene set (*p =* .0037) (see Supplement 1 for details). Additionally, the enrichment observed for sodium channels was significantly greater (*p =* .016) than random sets of genes sampled from all targeted paralogous genes (i.e., including sodium channels among the genes randomly sampled). An enrichment with a similar effect size was also observed after the exclusion of LoF variants (case-control meta-analysis: OR, 1.16; 95% CI, 1.04–1.29; *p =* .007), discounting the possibility that the additonal evidence provided by our analysis of paralog-conserved sites in sodium channels was merely a representation of the earlier primary finding of a nominal enrichment for LoF variants. As a further control for sequence quality, we found that the effect size for rare (frequency <0.1%) paralog-conserved missense and LoF variants was significantly different from that for paralog-nonconserved missense variants (*Z*-test *p =* .0018); indeed, as a negative control, there was no enrichment for missense variants at paralog-nonconserved sites (case-control meta-analysis: *p =* .44) (Figure 1, Table S5 in Supplement 2).

To dissect the voltage-gated sodium channel association, we divided the genes into their two primary functional groupings, alpha (10 genes) and beta (four genes) subunits, testing these separately. Only the alpha subunits were significantly enriched for rare (frequency <0.1%) paralog-conserved missense and LoF variants (case-control meta-analysis: alpha subunits, OR, 1.2; 95% CI, 1.08–1.33; *p =* .00086; *p*_*corrected*_ = .01; beta subunits, OR, 0.92; 95% CI, 0.52–1.62; *p*_*uncorrected*_ = .76). In all sodium-channel genes, a single nonsense de novo mutation was observed, that being in *SCN2A* (de novo *p* value for LoF and paralog-conserved missense variants in sodium-channel alpha subunits = .75; case-control-de novo meta-analysis: *p =* .0029; *p*_*corrected*_ = .035).

Paralog-conserved analysis did not reveal association with schizophrenia for individual voltage-gated sodium channel genes at the level required to demonstrate association (i.e., exome-wide significance) (Table S6 in Supplement 2) or even after adjusting for the experiment-wide context of 187 genes, although *SCN7A* showed a nominal signal (*p*_*uncorrected*_ = .001).

#### Primary Analysis of ARC and NMDAR Gene Sets

**Figure 2:**
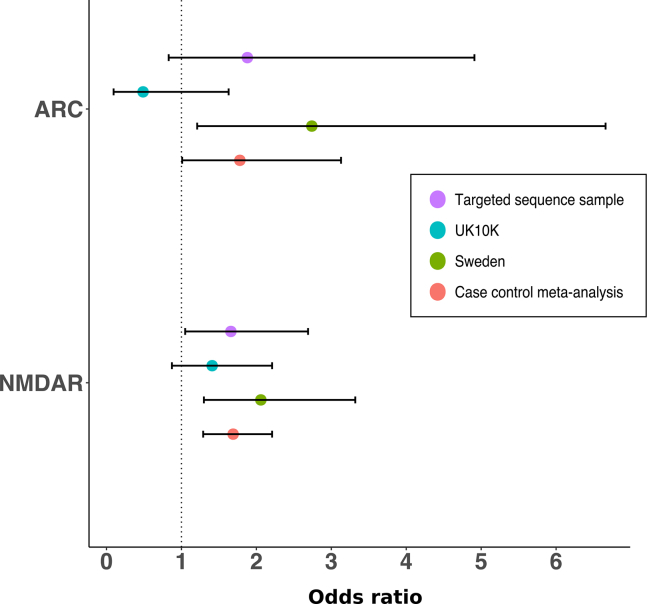
Case-control analysis of rare (frequency <0.1%) loss-of-function variants in activity-regulated cytoskeleton-associated protein (ARC) and *N*-methyl-D-aspartate receptor (NMDAR) synaptic gene sets (*n* = 28 and 61 genes, respectively). The case control meta-analysis comprises data from the targeted sequence sample (5207 cases and 4991 controls), the Sweden sample (4765 cases and 6107 controls), and the UK10K sample (1347 cases and 4756 controls).

In the targeted sequencing sample, cases had a higher rate of LoF variants (frequency <0.1%) in ARC (*p*_*uncorrected*_ = .14; OR, 1.88; 95% CI, 0.83–4.91) and NMDAR (*p*_*uncorrected*_ = .03; *p*_*corrected*_ = .12; OR, 1.66; 95% CI, 1.05–2.69) sets (Figure 2). When meta-analyzed with published case-control datasets, we found strong evidence that LoF variants in NMDAR complex genes were associated with schizophrenia (*p* = 1.6 × 10^–4^) (Figure 2 and Table S4 in Supplement 2), but weaker evidence for association with ARC complex genes (*p =* .047) (Figure 2 and Table S4 in Supplement 2).

To summarize the current status of RCVs in the above gene sets, we combined the case-control meta-analysis data with the de novo variant data, selecting the class of de novo data reported to be most strongly enriched in these gene sets (nonsynonymous de novo variants in ARC and LoF de novo variants NMDAR) in previous work (9). In the trio data, nonsynonymous and LoF de novo variants were associated with ARC (*p =* .0015) and NMDAR (*p =* .014), respectively. Combining the de novo enrichment results with the case-control meta-analysis results (LoF; frequency <0.1%), both ARC (*p* = 4.0 × 10^–4^) and NMDAR (*p* = 1.7 × 10^–5^) complexes were associated with schizophrenia (Table 2).

**Table 2:** Synaptic Gene Set Meta-analysis

Gene Set	Case-Control Meta-analysis				De Novo Analysis	Case-Control–De Novo Combined
	Mutations (Cases/Controls)	Rate (Cases/Controls)	*p* (Two-Sided)	OR (95% CI)	*p* (Observed/Expected)	*p* (One-Sided, Fisher’s Combined)
ARC (*n =* 28)	32/27	0.0028/0.0017	.047	1.78 (1.01–3.13)	.0015 (7/1.64)	4.0 × 10^–4^
NMDAR (*n =* 61)	114/111	0.01/0.007	.00016	1.69 (1.29–2.21)	.014 (3/0.49)	1.7 × 10^–5^

The case-control meta-analysis tested loss-of-function (LoF) variants (frequency <0.1%) for activity-regulated cytoskeleton-associated protein (ARC) and *N*-methyl-D-aspartate receptor (NMDAR) in 11,319 schizophrenia cases and 15,854 controls. The de novo analysis tested nonsynonymous and LoF variants in ARC and NMDAR in 1136 schizophrenia trios. Full details of the analysis are presented in Table S4 in Supplement 2. We note that the *p* values reported here are uncorrected (see Methods and Materials for rationale).

CI, confidence interval; OR, odds ratio.

The ARC and NMDAR complexes share nine overlapping genes: when excluded from the analysis, we observed independent evidence for association with both gene sets (case-control–de novo meta-analysis (ARC: *p* = 9.4 × 10^–4^; NMDAR: *p* = 7.4 × 10^–5^).

### Single-Gene Analysis

In the primary meta-analysis (LoF; frequency <0.1%) of all data, no gene was associated with schizophrenia after Bonferroni correction (Table S7 in Supplement 2). The most significant gene was *TAF13* (*p* = 1.6 × 10^–5^), with support coming mainly from published LoF de novo variants as noted before (9).

## Discussion

Sequencing studies have started to provide novel insights into the genetic architecture and etiology of schizophrenia, although these are still limited by small sample sizes and low power. Seeking to increase power for a prioritized set of genes, we sequenced the coding regions of 187 schizophrenia candidates in over 10,000 samples that have not contributed to previous sequencing studies of schizophrenia.

Across all candidates, we found a significant excess of LoF variants in the independent samples, confirming our hypothesis that one or more of the candidates is involved in schizophrenia pathogenesis. The strongest evidence for enrichment was for LoF variants with a frequency <0.1%, suggesting that recurrent rather than only singleton schizophrenia risk variants are present among our 187 targeted genes. This appears to contrast with a Swedish exome-sequencing study of schizophrenia, which reported an increased exome-wide burden in cases of ultra-rare protein altering variants observed only once in their sample and never in 45,376 nonpsychiatric Exome Aggregation Consortium individuals (7). Our analyses of the same Swedish dataset (Table S10 in Supplement 1) agrees with the primary study that at the exome-wide level, singleton LoF variants are more highly enriched than recurrent variants with a frequency <0.1% (*Z*-test *p =* .00035). However, this did not hold when restricted to the 187 targeted genes (*Z*-test *p =* .11) we have selected. Evidence for nonsingleton variants’ being enriched among specific sets of genes has been demonstrated in a recent analysis of the same Swedish data (35).

RCVs were enriched in our targeted genes with modest effect sizes when compared with specific rare variants previously associated with schizophrenia (e.g., CNVs). This may be a consequence of including variants in our burden analyses that are not related to schizophrenia, thus underestimating the effect size of causal variants. This limitation is inherent in sequencing studies and will only be overcome when true risk variants are known.

Among our sequenced genes were 14 voltage-gated sodium channels, which as a set were previously associated with schizophrenia in an analysis of parent-proband trios for compound heterozygous mutation, although this did not replicate (17). Rare variants in sodium channels have been associated with additional neurodevelopmental disorders, including some forms of epilepsy and developmental delay 18, 36, 37, which gives high plausibility that variants in these genes could also increase risk of schizophrenia. Given equivocal findings from previous studies implicating sodium channels in schizophrenia (17), our results provide novel evidence for association between RCVs in sodium channels and schizophrenia. We provide evidence that both LoF and missense variants at paralog-conserved sites in sodium channels increase risk of schizophrenia. This supports previous work that showed that paralog conservation scores can effectively identify missense variants associated with neurodevelopmental disorders (18).

The sodium channel set contains 14 genes–10 encoding alpha subunits involved in generating action potentials (36), and 4 beta subunits that, in association with alpha subunits, modulate their gating and cellular excitability (37). In our analysis, the evidence for association derives from variants in alpha subunits, although the absence of signal in beta subunits might simply reflect low power (there are fewer beta subunits, of which paralog conservation scores are only available for *SCN2B* and *SCN4B*, whereas paralog conservation scores are available for all 10 alpha subunits).

The statistical evidence we report for association with sodium channels survived a study-wide Bonferroni correction for multiple testing, was robust to permutation testing, and has high plausibility in the context of sodium-channel associations in other neurodevelopmental disorders; nevertheless, despite our use of virtually all published sequencing data that are publicly available, it will be necessary for future studies to confirm this before the finding can be considered definitive.

In the present study, we conducted the largest schizophrenia sequencing meta-analysis of RCVs in the ARC and NMDAR synaptic gene sets to date. The inclusion of our new independent data in this analysis strengthened the evidence for association between RCVs in ARC and NMDARs and schizophrenia. In the context of previously published research, in which rare and de novo CNVs in these gene sets have been consistently associated with schizophrenia 5, 10, 14, the results now provide a strong and consistent body of evidence for the involvement of ARC and NMDAR proteins in the etiology of schizophrenia.

Despite the increased sample size, we did not observe any single-gene association that was significant at a genome-wide significant level, or even a study-wide level, and therefore it is not possible to infer causal associations between any of the variants, or genes, presented in this study. Doing so will require even larger samples, and possibly other methods for classifying missense variation.

In our new targeted sequence sample, ∼81% of cases were from the CLOZUK cohort, a cohort of individuals whose phenotype comprises a clinician reported diagnosis of treatment-resistant schizophrenia (TRS) requiring clozapine treatment. The CLOZUK cohort has been previously validated [see supplemental note in (4)] and has a similar common and CNV variant architecture to schizophrenia samples diagnosed using research instruments (6). This sample is likely to be overrepresented for certain features including increased severity, poorer cognition, early onset, and (by definition) treatment resistance, but we are unable to examine the impact these phenotypes may have had on our results. Therefore, it is possible that our findings reflect association with those phenotypic aspects of the disorder rather than liability in general. Moreover, as many of our controls (*N =* 2463) are blood donors, these are likely to be psychiatrically healthier than the general population. These sampling frameworks enhance power for discovery, but a corollary is that it is likely to inflate effect sizes, so follow-up studies in general population samples are required.

Differences in allele frequencies caused by phenotypes associated with TRS would most likely be observed in LoF-intolerant genes, given their consistent association with severe neurodevelopmental phenotypes 16, 38. However, we find no evidence of heterogeneity in our case-control meta-analysis of rare, LoF variants in all 106 sequenced LoF-intolerant genes (Cochran’s *Q* = 1.23, *p =* .54). Nonetheless, deep phenotyping of individuals carrying schizophrenia risk variants and investigating differences in the risk conferred by rare variants between TRS and non-TRS are important areas for future research. Additional limitations in our study include the exclusion of indel mutations (see Supplement 1) from the targeted sequencing data, and the inability to test some of the larger gene sets that have been implicated in schizophrenia (e.g., fragile X mental retardation protein targets).

In conclusion, we conducted one of the largest sequencing studies of schizophrenia to date, which targeted the protein coding regions of 187 putative schizophrenia risk genes. By leveraging information from paralog conservation, we provide novel evidence that multiple voltage-gated sodium channels are involved in schizophrenia pathogenesis. We provide further support for association between RCVs in ARC and NMDAR postsynaptic protein complexes and schizophrenia. While it is premature to speculate on the mechanistic and therapeutic implications of the current findings, we note the implication of sodium-channel genes adds to evidence, including previous work implicating postsynaptic protein complexes, pointing to fundamental abnormalities of neuronal activity in schizophrenia as well as suggesting the possibility that these may be tractable to novel and existing pharmacological approaches.
