# Supplementary material for: Targeted Sequencing of 10,198 Samples Confirms Abnormalities in Neuronal Activity and Implicates Voltage-Gated Sodium Channels in Schizophrenia Pathogenesis
Source: Biol Psychiatry. 2019 Apr 1;85(7):554–62. doi: 10.1016/j.biopsych.2018.08.022 (PMC6428681; doi:10.1016/j.biopsych.2018.08.022)
Supplement: Supplemental Material [file mmc1.pdf]

Targeted Sequencing of 10,198 Samples Confirms Abnormalities in Neuronal Activity and Implicates Voltage-Gated Sodium Channels in Schizophrenia Pathogenesis

Supplement 1

Table of Contents

Legends for Excel supplementary tables 2
Sample description 4
Published trio data sets used to derive schizophrenia *de novo* variants 5
Targeted sequencing protocol 6
Gene selection criteria 8
Data processing and quality control 9
Variant annotation 12
Statistics 13
Approach to hypothesis testing and multiple testing 16
Analysis of singleton vs. rare (frequency < 1%), non-singleton, LoF variants in the Swedish exome sequencing sample 19
Power calculations 20
References 21

Legends for Excel supplementary tables

See Supplement 2 (Excel file) for tables

Table S1. Genes targeted for sequencing. Gene IDs are presented for 187 targeted genes, along with the criteria used to select them for sequencing.

Table S2. Targeted sequence sample case-control LoF variants. All loss of function (LoF) variants observed in the new targeted sequence data, for alleles < 0.1% in frequency. Singletons are indicated in the 'Is Singleton' column. The 'DDG2P Gene' column indicates whether the affected gene is a confirmed developmental disorder gene associated with monogenic LoF variants (defined using data downloaded from DECIPHER (https://decipher.sanger.ac.uk/about#downloads/data) on 21/08/2018 (1)).

Table S3. Total variant burden analysis. Case-control association results for all 187 targeted genes and 106 LoF intolerant genes (genes with pLi scores > 0.9). The minor allele frequency threshold used for the given test is shown in the 'Variant frequency' column.

Table S4. Primary gene set analysis. Gene set association results for three case-control data sets (Targeted sequence sample, Swedish, UK10K) and case-control-*de novo* meta-analysis (Fisher's combined method). The minor allele frequency threshold used for the given test is shown in the 'Variant frequency' column.

Table S5. Secondary gene set analysis of LoF and paralog conserved missense variants (<0.1% frequency) in the ion channel gene sets. Paralog conservation scores (para_zscores) were downloaded from https://zenodo.org/record/817898.

Table S6. Single-gene meta-analysis of sodium channel genes for LoF and paralog conserved missense variants (<0.1% frequency).

Table S7. Primary single-gene meta-analysis of LoF variants (<0.1% frequency). Single-gene results for three case-control (Ion torrent, Swedish, UK10K) and de novo mutations.

Sample description

Cases

Targeted sequence sample: 5,724 schizophrenia cases (pre-QC) were sequenced using Ion Torrent instruments. The majority of these cases were from the CLOZUK cohort (n=4,647), which consists of individuals diagnosed with treatment resistant schizophrenia. Here, DNA was extracted from anonymised whole blood samples. All CLOZUK samples had received a clinician reported diagnosis of treatment-resistant schizophrenia. The CLOZUK cohort has been extensively used in previous common allele (2, 3) and rare CNV genetic studies (4, 5), and a validation for using a clinician diagnosis of treatment-resistant schizophrenia against a research diagnostic criteria for schizophrenia can be found in Pardiñas *et al* 2017, Supplementary Note (2). We sequenced additional cases from the UK belonging to the CardiffCOGs cohort (n=521). These cases were assessed with a SCAN interview (6) and case note review followed by consensus research diagnostic procedures. All CardiffCOGs cases had a DSM-IV diagnosis of schizophrenia or schizoaffective disorder-depressive type. Further details on the CardiffCOGs cohort can be found in previously published studies (2, 5).

335 cases were recruited from Ireland (Dublin cohort). These cases were all over 18 years of age and had a diagnosis of Schizophrenia or Schizoaffective Disorder after a structural clinical assessment (as described in (7)). Diagnosis was made based on the consensus lifetime best estimate method using all available information (interview, family or staff report, chart review) with DSM-IV criteria as per the Structured Clinical Interview for DSM-IV, research edition (SCID-P). Each referral centre obtained local Research Ethics Committee (REC) approval. 221 additional cases were recruited from the Netherlands (GROUP cohort). The GROUP cohort has been

described previously (8). Cases were between 16 and 50 years of age, and had received a diagnosis of schizophrenia according to DSM-IV criteria.

Controls

The majority of sequenced controls were part of the WTCCC2 consortium (1958 birth cohort n=2860, UK blood donors n=2463) (9-11). Additional controls were sequenced from the Dublin (n=230) and GROUP cohorts (n=216) (8).

Published trio data sets used to derive schizophrenia *de novo* variants

Published schizophrenia exome sequencing trio studies used to derive schizophrenia *de novo* mutations are shown in Table S8. We included data sets that reported all exome-wide nonsynonymous *de novo* mutations.

Table S8. Sources of published schizophrenia *de novo* variants.

Publication	N probands (Male:Female)
Fromer *et al* 2014 (12)	617 (302:315)
Girard *et al* 2011 (13)	14 (7:7)
Xu *et al* 2012 (14)/Takata *et al* 2014 (15)*	231 (156:75)
Gulsuner *et al* 2013 (16)	105 (75:30)
Wang *et al* 2015 (17)	45 (22:23)
Ambalavanan *et al* 2015 (18)	17 (11:6)
Guipponi *et al* 2014 (19)	53 (39:14)
McCarthy *et al* 2014 (20)	54 (43:11)

Studies that reported all nonsynonymous *de novo* variants discovered from whole exome sequencing were included. * Takata *et al* 2014 (15) re-analysed the data published by Xu et al 2012 (14) to discover *de novo* indels missed in the original study.

Targeted sequencing protocol

We designed an Ampliseq custom panel (Thermo Fisher) for targeting the exons of 187 genes. The panel comprised two pools of 3,094 and 3,082 amplicons each and covered a total region of 750kb. Library preparation used the Ion AmpliSeq protocol, using 10ng of DNA per pool, the Ion Ampliseq Kit Version 2.0 and the AmpliSeq custom panel (Thermo Fisher). We barcoded the libraries using the Ion Express Barcode Adapters 1-96 Kit (Thermo Fisher). Unamplified libraries were quantified by qPCR using Ion TQMN Quantitation kit following manufacturer's instructions (Thermo Fisher) and diluted to 100pM. Groups of 72 uniquely barcoded libraries were combined onto single Ion Chips. Sequencing was performed on the Ion Proton benchtop sequencing platform (Thermo Fisher) following the manufacturer's protocol. Sequencing took place in two waves, corresponding to different versions of the sequencing and chip kits: 2,305 cases and 2,274 controls were sequenced in wave 1 using the Ion PI IC 200 kit and Ion PI Chip kit v2 BC (Thermo Fisher); 3,419 cases and 3,495 controls were sequenced in wave 2 using the Ion PI HiQ kit and Ion PI Chip kit v3 BC (Thermo Fisher). Data processing and QC procedures were conducted independently for each wave. The mean target coverage for cases and controls passing QC was 158X and 160X for wave 1, and 154X and 145X for wave 2, respectively (density plots of sequence coverage in Supplementary Material Fig S1). Both cases and controls had at least 95% of target bases covered at ≥10X.

Sequencing coverage metrics

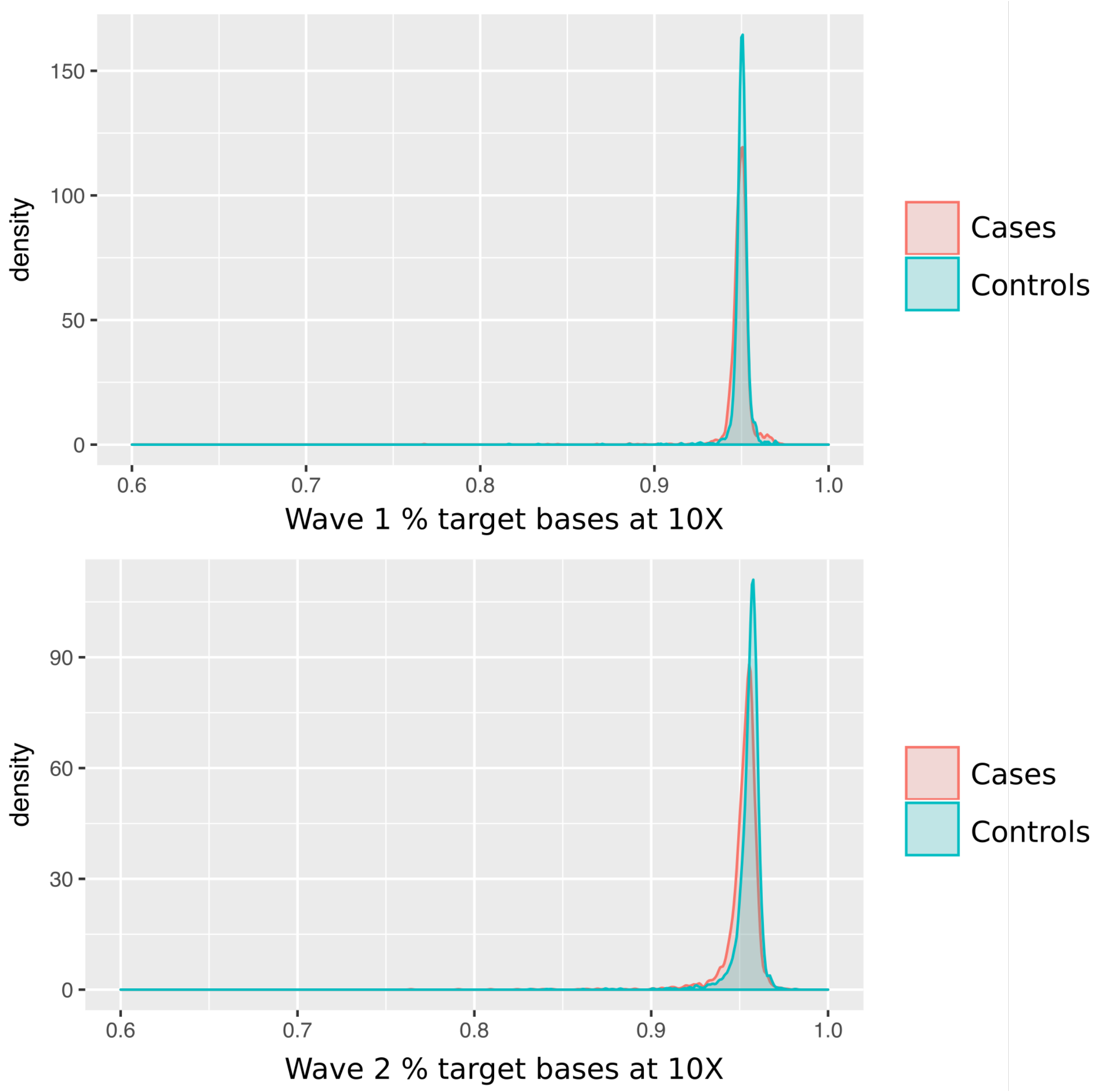

Fig S1. Density plots showing the % of target bases covered at ≥10 X in targeted sequencing waves 1 and 2.

Gene selection criteria

We sequenced the coding regions of 187 genes, 129 of which belong to four gene-sets that have been previously implicated in schizophrenia ('Schizophrenia-associated biological gene-sets' selection criteria in Table S9). We also sequenced 58 additional genes that have at least two lines of evidence for association with schizophrenia. These lines of evidence were based on gene-sets previously implicated in schizophrenia, for example FMRP (21) and miR-137 (3) targets, genes disrupted by *de novo* SNVs/indels in patients diagnosed with schizophrenia, autism spectrum disorder or intellectual disability (12), genes within schizophrenia-associated CNV loci (5) and genes disrupted by schizophrenia *de novo* CNVs (22) (full list of criteria described in Table S1).

Table S9. Criteria for selecting genes for Ion torrent sequencing.

**Gene-set**	**N genes in set**	**N genes Sequenced in current study**	**% of gene-set sequenced**	**Selection criteria**
ARC	28	28	100	Schizophrenia-associated biological gene-sets.
NMDAR	61	61	100	
VGSC	14	14	100	
VGCC	26	26	100	
Post-synaptic density	664	84	12.7	Additional gene-sets implicated in schizophrenia.
SZ Nonsynonymous de novo	611	58	9.5	
SZ LoF de novo	87	21	24.1	
ID Nonsynonymous de novo	132	16	12.1	
ID LoF de novo	30	5	16.6	
ASD Nonsynonymous de novo	743	39	5.2	
ASD LoF de novo	128	7	5.5	
FMRP target	786	60	7.6	

**Gene-set**	**N genes in set**	**N genes Sequenced in current study**	**% of gene-set sequenced**	**Selection criteria**
SZ de novo CNV	228	10	4.4	
mGluR5	39	15	38.5	
miR-137 target	446	14	3.1	
PGC GWAS hit	473	45	9.5	
SZ-associated CNV	269	4	1.5	
Recurrent SZ de novo SNV	49	32	65.3	

All genes belonging to ARC, NMDAR, Voltage-gated calcium channels (VGCCs) and voltage-gated sodium channels (VGSCs) gene-sets were sequenced (rows above dashed line), as well as genes that were members of two or more gene-sets listed below the dashed line.

Data processing and quality control

Sequence data were independently processed for each Ion Torrent wave according to GATK best practice guidelines (23, 24). Reads were aligned to the human reference genome (GRCh37) using bwa (25). Variants were called using GATK haplotype caller (v3.4) and filtered using the GATK Variant Quality Score Recalibration (VQSR) tool.

Sample level QC: Individuals were excluded if they were more than 3 standard deviations from their sequencing wave's mean for: proportion of variants in dbSNP; number of alternative alleles; number of singletons; total number of synonymous mutations; total number of nonsynonymous mutations. When available, SNP genotyping array data were used to assess sequencing-array genotype concordance (array genotypes used as truth set) and to identify duplicate/first degree relatives. SNP genotyping array data from 3 chips (Illumina OmniExpress, Illumina ExomeChip,

Immunochip) were available for 96% (5,508/5,724) of cases and 72% (4,149/5,769) of controls. Samples were excluded if they had a genotype concordance < 0.9 or if they were found to be one member of a duplicate (kinship coefficient > 0.354) or 1st degree relative (kinship coefficient > 0.177) pair (identified using the KING toolset (26) in the Bioconductor package SNPRelate). For samples not previously genotyped using SNP arrays, we used Ion Torrent sequence data to identify and exclude duplicate samples.

Principal component analysis (PCA) was used to identify and exclude cases and controls with non-European ancestry. We performed PCA in the 1000 genomes project data (phase 3), using variants found in both targeted sequence data and 1000 genomes data, and projected our targeted sequence samples onto these PCs using EIGENSOFT smartPCA (27). Targeted sequence samples were excluded if PCs 1 and 2 were more than 3 standard deviations from the mean of PCs from European 1000 genome samples (Supplementary Fig S2). Post sample QC, 5,207 cases and 4,991 controls from the targeted sequence sample were retained for analysis.

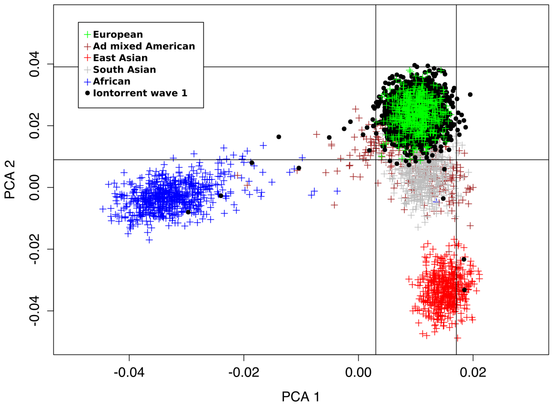

A

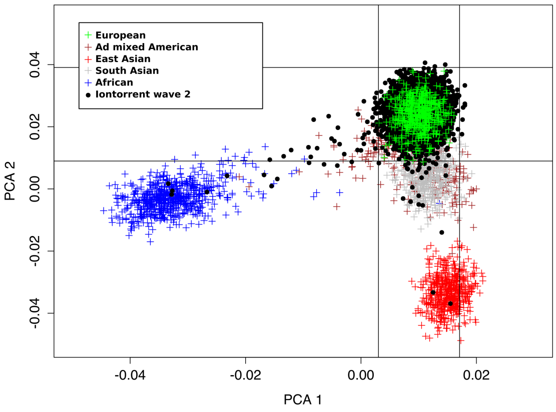

B

Fig S2. Identifying sample ancestry through principle component analysis **A.)** targeted sequence samples sequenced in wave 1. **B.)** targeted sequence samples sequenced in wave 2. Vertical and horizontal lines in **A** and **B** indicate 3 standard deviations from the mean PCs from European 1000 genome European samples, which were the thresholds used to exclude non-European samples from the case-control analysis.

Variant level QC: Variant sites within each targeted Ion Torrent sequencing wave were excluded if they failed Hardy-Weinberg equilibrium exact tests (χ^2 $P < 10^{-8}$), GATK VQSR filters, had > 20% missingness, or contained an indel (sequence data produced by Ion Torrent instruments has low indel specificity (28)). For targeted sequencing data, individual genotypes were set to missing if they had a depth (DP) < 20, genotype quality (GQ) < 80, allele-balance (AB) < 0.9 for non-reference

homozygous genotypes or an AB < 0.2 or > 0.8 for heterozygous genotypes. For analysis of previously published exome sequencing data, we applied filters which more closely matched those described in their original publications (DP <=10, GQ < 30, AB < 0.9 for non-reference homozygous genotypes, AB < 0.2 or > 0.8 for heterozygous genotypes) (29, 30). All variant filtering was conducted using Hail software (https://github.com/hail-is/hail) (31).

Variant annotation

In primary burden tests, we analysed three classes of mutation (LoF, nonsynonymous damaging, and nonsynonymous) and two allele frequency thresholds (<0.1% and singletons). We defined LoF variants as those producing premature stop codons (nonsense) or situated at essential splice sites (within 2 bases either side of exon junctions). Damaging nonsynonymous mutations were defined as LoF variants and missense variants with a PHRED-scaled CADD score ≥ 20 (representing the predicted top 1% most deleterious variants in the genome) (32). For analysis of previously published case-control data (UK10K and Swedish samples), which were exome sequenced on Illumina instruments, we included frameshift indels as LoF mutations and frameshift/in-frame indels as nonsynonymous mutations. We annotated and filtered variants using the frequencies observed in their respective data set (Ion Torrent wave 1, Ion Torrent wave 2, UK10K or Swedish) and each ExAC sub-population (European (Non-Finnish), African, East Asian, European (Finnish), Latino, Other, South Asian) (33). Singletons were annotated as variants observed once in all available sequence data (targeted, UK10K and Swedish data) and never in 45,376 individuals without a known psychiatric diagnosis from the Exome Aggregation Consortium (33).

To annotate sites with their paralog conservation scores, we used para_zscores downloaded from https://zenodo.org/record/817898. In our paralog conserved analysis,

we followed the publication describing this metric (34) by testing the burden of all LoF variants and missense variants at sites annotated as having a para_zscore > 0. Variants were annotated using Hail's ensemble VEP method (version 86, http://oct2016.archive.ensembl.org/index.html).

Statistics

Case-control analysis: Gene set and single gene association statistics were generated using Firth's penalised-likelihood logistic regression model as follows:

> Logit (pr(case)) ~ N test variants + baseline synonymous count + first 10 PCs + sex + Ion Torrent sequencing wave (targeted analysis only).

N test variants refers to the number of rare variants observed in each sample (e.g. number of LoF singletons in the gene/gene set tested). Baseline synonymous count refers to the number synonymous variants observed in all sequenced genes, using the same allele frequency threshold used for the test variant (e.g. if LoF singletons are the test variant, then the overall number of synonymous singletons are corrected for). This covariate was only included in tests of nonsynonymous, LoF, or paralog conserved missense variants to control for potential unknown technical biases (29).

Test statistics were generated independently for each case-control dataset (targeted, UK10K and Swedish), using the logistf function implemented in R (version 3.3.1). Odds ratios (ORs) for the increased risk of schizophrenia incurred for each mutation were obtained from the Firth's penalised-likelihood logistic regression model described above.

Simulation analysis: We compared *P* values obtained from logistic regression models (i.e. the primary statistical test) to those generated from randomly permuted data. Here, logistic regression tests were repeated with randomised case/control status, and significance was determined as the fraction of permuted tests as, or more, significant than the original *P* value.

To assess whether exome sequencing case-control analyses are controlled for unknown confounds, recent studies have shown that enrichment *P* values for sets of genes randomly sampled across the exome follow the expected null distribution (e.g. Singh et al 2017 (35)). However, our study design precludes the use of random gene sets as a means for testing null distributions, as the genes are, by design, selected as candidate genes for schizophrenia. Nevertheless, we tested the enrichment of sodium channel genes for LoF and paralog conserved missense variants in schizophrenia by comparing its significance to that observed from 100,000 random sets of 14 genes (the number of genes in our voltage-gated sodium channels gene set), sampled from our 137 targeted genes that had paralog conserved sites. We performed two random gene set tests: the first excluded sodium channels from the random gene draws to test whether the sodium channel result reflected a general increased burden for LoF variants and missense variants at paralog sites across all targeted genes; the second included sodium channels among the random gene draws to test whether the enrichment is due to the genes being sodium channels, as opposed to being other pathways containing the most significant genes.

Significance was determined as the fraction of random gene sets as, or more, significant than the original sodium channel *P* value. This is likely to be a more conservative test than comparing sodium channels to random gene sets selected from

the whole genome, since one would expect the 187 schizophrenia candidate genes as a whole to show an excess of LoF variants in schizophrenia.

Analysis of *de novo* variants: Enrichment of *de novo* variants in genes/gene sets was tested using the statistical framework described in Samocha *et al* 2014 (36). Here, gene mutation rates provided in Ware *et al* 2015 (37) were used to estimate the expected number of *de novo* mutations in the gene/gene set, which was then compared to the observed number of *de novo* mutations using a Poisson test (implemented in R). As gene mutation rates for in-frame indels are not provided in Ware *et al* 2015 (37), we adopted the method used by the Deciphering Developmental Disorders Study (38), which scaled frameshift mutation rates by the ratio of in-frame to frameshift mutations reported to occur in genome-wide regions under weak negative selection (ratio 1:9) (38). To estimate the expected number of LoF *de novo* mutations and missense *de novo* mutations at paralog conserved sites in sodium channel alpha subunits, we used the mutation rates provided in Lal *et al* 2017 (34).

Case-control *de novo* meta-analysis: Coefficients and standard errors from independent case-control regression tests (targeted, UK10K and Swedish) were meta-analysed as fixed effects using the inverse-variance method (implemented in R using the rma.uni() function as part of the metafor package). To obtain a single enrichment statistic for meta-analysed case-control and *de novo* tests, we followed the method described in Singh *et al* 2016 (30), which combined a 1-tail case-control *P* value with the *de novo* Poisson test *P* value using Fisher's combined method. For combined case-control-*de novo* meta-analysis of nonsynonymous damaging mutations, we included all *de novo* nonsynonymous mutations (i.e. not just those with a CADD score ≥ 20), given

they are *a priori* more likely to be deleterious than inherited variation (39) and were the class of mutation most strongly associated with schizophrenia candidate genes in our previous publication (12).

Approach to hypothesis testing and multiple testing

Our targeted sequencing study was designed to test three broad questions:

1. Do we observe significant evidence for enrichment of rare variants in 187 genes previously implicated in schizophrenia?
2. Do we support enrichment of rare variants in four candidate gene-sets previously implicated in schizophrenia?
3. Do we observe significant evidence for enrichment of rare variants in single genes?

For each of these questions, we describe below our rationale for the classes of variant tested and the number of multiple tests.

1. Do we observe significant evidence for enrichment of rare variants in 187 genes previously implicated in schizophrenia?

Given our targeted genes had different lines of evidence implicating them in schizophrenia, we had no strong prior hypothesis for a specific class of mutation being enriched among cases. Therefore, we performed 6 burden tests to cover three classes of mutation (LoF, nonsynonymous damaging, and nonsynonymous) and two allele frequency thresholds (<0.1% and singletons) (main text Table 1). For enrichment analysis of nonsynonymous damaging variants, we used CADD scores to define

damaging missense variants, as this metric is widely used in exome-sequencing studies and is available for all genes. We did not use paralog scores in our enrichment analysis of all 187 genes (paralog scores tested below under Secondary gene-set analysis), as this metric was not available when we first specified our study design, and moreover, the set of 187 genes do not comprise a functionally related gene set where it could be postulated that domains that are conserved across members might be disease relevant. The *P*-values derived from the enrichment tests in the full set of 187 genes were **Bonferroni corrected for 6 tests.** For mutation classes significant in the primary burden tests, we performed a single analysis to characterize the enrichment, by partitioning the targeted genes into those intolerant of LoF variants (pLi > 0.9) and those that are not (pLi ≤ 0.9). Since this single analysis was designed to further characterize the source of an association signal, no multiple testing correction was applied.

2. Do we support enrichment for rare variants in four candidate gene-sets previously implicated in schizophrenia?

Primary gene-set analysis*:* Among the targeted genes are four gene-sets previously implicated in schizophrenia; these include the synaptic sets ARC (n=28) and NMDAR (n=61) (12), and the ion-channel sets voltage-gated calcium channels (n=26) (21) and voltage-gated sodium channels (n=14) (40). Given rare (< 0.1% frequency), LoF variants was the only class of mutation from question 1 that survived correction for multiple testing (main text Table 1), we tested these variants in a primary gene-set analysis. In the new targeted sequencing sample, *P* values from the primary gene-set analysis were **Bonferroni corrected for 4 tests** (four gene-sets x one mutation class).

For meta-analysis, we note the inclusion of ARC, NMDAR, and calcium channel gene sets in the present study was predicated on previous associations from exome-wide *de novo* and case-control studies that are included in the present meta-analysis (12, 21). This ascertainment bias makes it impossible to generate meaningful and appropriately conservative study-wide multiple-testing corrections. We therefore consider those meta-analyses as representing an appraisal of the current sequencing evidence for those gene-sets. The case-control meta-analysis of sodium channels does not include any previously reported data, and therefore it does not suffer from such an ascertainment bias; accordingly, we calculate study-wide corrected *P*-values as we did for the new sequencing data (four gene-sets x one mutation class).

Secondary gene-set analysis: The two ion-channel gene sets are largely comprised of paralogous genes; therefore, we leveraged information from a recently described metric of nucleotide-paralog conservation to test them under a secondary definition of nonsynonymous damaging mutations, which included LoF variants and missense variants disrupting paralog conserved sites. Although we only performed this secondary analysis for the two ion channel gene-sets, *P* values in our secondary gene-set analysis were **Bonferroni corrected for potentially testing all four gene-sets under primary (LoF) and secondary (LoF + paralog conserved missense) mutation classes (8 tests).**

We performed a number of exploritory tests to assess the robustness of the enrichment of LoF and paralog conserved missense variants in sodium channels, and to dissect which genes might be driving the signal. In this later test, we partioned sodium channel genes into alpha and beta subunits. Aiming to favour caution in the light of the novelty of the finding, we conservatively **Bonferroni corrected the**

derived *P*-values for 12 potential tests (two mutation classes tested against four gene-sets plus the two sub-sets of sodium channels alpha and beta subunits).

3. Do we observe significant evidence for enrichment of rare variants in single genes?

Single gene analysis: Our primary single-gene enrichment analysis tested rare (< 0.1% frequency), LoF variants, again as this was the only class of mutation from question 1 that survived correction for multiple testing. In our meta-analysis, we applied exome-wide criteria for multiple testing correction by applying **Bonferroni correction for testing 20,000 genes.**

Analysis of singleton vs. rare (frequency < 1%), non-singleton, LoF variants in the Swedish exome sequencing sample

To test our hypothesis that our 187 targeted genes are more likely to contain rare (frequency < 1%), non-singleton schizophrenia risk alleles compared with all genes, we compared effect sizes between these tests. As expected, we reproduced the published Swedish result (29) in finding a significant effect size difference between singleton and rare (frequency < 1%), non-singleton tests at the exome-wide level (Table S10). However, no significant difference was observed when the analysis was restricted to the 187 targeted genes (Table S10), suggesting the existence of risk alleles within these genes that have a minor allele count > 1.

Table S10. Case-control analysis of singleton and rare (frequency <0.1%, excluding singletons) LoF variants in the Swedish exome sequencing data set.

Test	Frequency Singletons		Frequency <0.1% (excluding singletons)		Z-test p-value of effect size difference
	P	OR (95% CI)	P	OR (95% CI)	
Exome-wide	0.000012	1.07 (1.04-1.1)	0.43	1.0072 (0.99-1.03)	0.00035
187 targeted genes	0.0086	1.44 (1.1 – 1.9)	0.17	1.16 (0.94-1.44)	0.11

Power calculations

Table S11. Power calculation. For the sample size used in the targeted sequencing sample (5,207 cases, 4,991 controls) and the case-control meta-analysis (11,319 cases, 15,854 controls), power calculations (41) are provided for risk alleles present at 1% cumulative frequency, with an effect size of 1.5, and an α which corrects for multiple testing in the given test.

Test	α	Power	
		Targeted sequencing sample	Meta-analysis sample
Primary burden	0.0083	0.70	0.99
Primary gene-set	0.0125	0.75	0.99
Secondary gene-set	0.0042	0.62	0.98
Single-gene	2.5×10^{-6}	0.062	0.62

References

1. Firth HV, Richards SM, Bevan AP, Clayton S, Corpas M, Rajan D, et al. (2009): DECIPHER: Database of Chromosomal Imbalance and Phenotype in Humans Using Ensembl Resources. *The American Journal of Human Genetics*. 84:524-533.
2. Pardiñas AF, Holmans P, Pocklington AJ, Escott-Price V, Ripke S, Carrera N, et al. (2018): Common schizophrenia alleles are enriched in mutation-intolerant genes and in regions under strong background selection. *Nat Genet*. 50:381-389.
3. Schizophrenia Working Group of the Psychiatric Genomics Consortium (2014): Biological insights from 108 schizophrenia-associated genetic loci. *Nature*. 511:421-427.
4. Rees E, Kendall K, Pardiñas AF, Legge SE, Pocklington A, Escott-Price V, et al. (2016): Analysis of intellectual disability copy number variants for association with schizophrenia. *JAMA Psychiatry*. 73:963-969.
5. Rees E, Walters JTR, Georgieva L, Isles AR, Chambert KD, Richards AL, et al. (2014): Analysis of copy number variations at 15 schizophrenia-associated loci. *Br J Psychiatry*. 204:108-114.
6. Wing JK, Babor T, Brugha T, Burke J, Cooper JE, Giel R, et al. (1990): SCAN: Schedules for Clinical Assessment in Neuropsychiatry. *Arch Gen Psychiatry*. 47:589-593.
7. Irish Schizophrenia Genomics Consortium and the Wellcome Trust Case Control Consortium 2 (2012): Genome-Wide Association Study Implicates HLA-C*01:02 as a Risk Factor at the Major Histocompatibility Complex Locus in Schizophrenia. *Biol Psychiatry*. 72:620-628.
8. Korver N, Quee PJ, Boos HBM, Simons CJP, de Haan L, investigators G (2012): Genetic Risk and Outcome of Psychosis (GROUP), a multi site longitudinal cohort study focused on gene–environment interaction: objectives, sample characteristics, recruitment and assessment methods. *Int J Methods Psychiatr Res*. 21:205-221.
9. Power C, Atherton K, Strachan DP, Shepherd P, Fuller E, Davis A, et al. (2007): Life-course influences on health in British adults: effects of socio-economic position in childhood and adulthood. *Int J Epidemiol*. 36:532-539.
10. Power C, Elliott J (2006): Cohort profile: 1958 British birth cohort (National Child Development Study). *Int J Epidemiol*. 35:34-41.
11. Wellcome Trust Case Control Consortium (2007): Genome-wide association study of 14,000 cases of seven common diseases and 3,000 shared controls. *Nature*. 447:661-678.
12. Fromer M, Pocklington AJ, Kavanagh DH, Williams HJ, Dwyer S, Gormley P, et al. (2014): De novo mutations in schizophrenia implicate synaptic networks. *Nature*. 506:179-184.
13. Girard SL, Gauthier J, Noreau A, Xiong L, Zhou S, Jouan L, et al. (2011): Increased exonic de novo mutation rate in individuals with schizophrenia. *Nat Genet*. 43:860-863.

14. Xu B, Ionita-Laza I, Roos JL, Boone B, Woodrick S, Sun Y, et al. (2012): De novo gene mutations highlight patterns of genetic and neural complexity in schizophrenia. *Nat Genet*. 44:1365-1369.

15. Takata A, Xu B, Ionita-Laza I, Roos JL, Gogos Joseph A, Karayiorgou M (2014): Loss-of-Function Variants in Schizophrenia Risk and SETD1A as a Candidate Susceptibility Gene. *Neuron*. 82:773-780.

16. Gulsuner S, Walsh T, Watts AC, Lee MK, Thornton AM, Casadei S, et al. (2013): Spatial and temporal mapping of de novo mutations in schizophrenia to a fetal prefrontal cortical network. *Cell*. 154:518-529.

17. Wang Q, Li M, Yang Z, Hu X, Wu H-M, Ni P, et al. (2015): Increased co-expression of genes harboring the damaging de novo mutations in Chinese schizophrenic patients during prenatal development. *Sci Rep*. 5:18209.

18. Ambalavanan A, Girard SL, Ahn K, Zhou S, Dionne-Laporte A, Spiegelman D, et al. (2016): De novo variants in sporadic cases of childhood onset schizophrenia. *Eur J Hum Genet*. 24:944-948.

19. Guipponi M, Santoni FA, Setola V, Gehrig C, Rotharmel M, Cuenca M, et al. (2014): Exome Sequencing in 53 Sporadic Cases of Schizophrenia Identifies 18 Putative Candidate Genes. *PLoS ONE*. 9:e112745.

20. McCarthy S, Gillis J, Kramer M, Lihm J, Yoon S, Berstein Y, et al. (2014): De novo mutations in schizophrenia implicate chromatin remodeling and support a genetic overlap with autism and intellectual disability. *Mol psychiatry*. 19:652-658.

21. Purcell SM, Moran JL, Fromer M, Ruderfer D, Solovieff N, Roussos P, et al. (2014): A polygenic burden of rare disruptive mutations in schizophrenia. *Nature*. 506:185-190.

22. Kirov G, Pocklington AJ, Holmans P, Ivanov D, Ikeda M, Ruderfer D, et al. (2012): De novo CNV analysis implicates specific abnormalities of postsynaptic signalling complexes in the pathogenesis of schizophrenia. *Mol Psychiatry*. 17:142-153.

23. McKenna A, Hanna M, Banks E, Sivachenko A, Cibulskis K, Kernytsky A, et al. (2010): The Genome Analysis Toolkit: A MapReduce framework for analyzing next-generation DNA sequencing data. *Genome Res*. 20:1297-1303.

24. DePristo MA, Banks E, Poplin R, Garimella KV, Maguire JR, Hartl C, et al. (2011): A framework for variation discovery and genotyping using next-generation DNA sequencing data. *Nat Genet*. 43:491-498.

25. Li H, Durbin R (2009): Fast and accurate short read alignment with Burrows–Wheeler transform. *Bioinformatics*. 25:1754-1760.

26. Manichaikul A, Mychaleckyj JC, Rich SS, Daly K, Sale M, Chen W-M (2010): Robust relationship inference in genome-wide association studies. *Bioinformatics*. 26:2867-2873.

27. Patterson N, Price AL, Reich D (2006): Population Structure and Eigenanalysis. *PLOS Genetics*. 2:e190.

28. Zhang G, Wang J, Yang J, Li W, Deng Y, Li J, et al. (2015): Comparison and evaluation of two exome capture kits and sequencing platforms for variant calling. *BMC Genomics*. 16:581.

29. Genovese G, Fromer M, Stahl EA, Ruderfer DM, Chambert K, Landen M, et al. (2016): Increased burden of ultra-rare protein-altering variants among 4,877 individuals with schizophrenia. *Nat Neurosci*. 19:1433-1441.

30. Singh T, Kurki MI, Curtis D, Purcell SM, Crooks L, McRae J, et al. (2016): Rare loss-of-function variants in SETD1A are associated with schizophrenia and developmental disorders. *Nat Neurosci*. 19:571-577.

31. Ganna A, Genovese G, Howrigan DP, Byrnes A, Kurki MI, Zekavat SM, et al. (2016): Ultra-rare disruptive and damaging mutations influence educational attainment in the general population. *Nat Neurosci*. 19:1563.

32. Kircher M, Witten DM, Jain P, O'Roak BJ, Cooper GM, Shendure J (2014): A general framework for estimating the relative pathogenicity of human genetic variants. *Nat Genet*. 46:310-315.

33. Lek M, Karczewski KJ, Minikel EV, Samocha KE, Banks E, Fennell T, et al. (2016): Analysis of protein-coding genetic variation in 60,706 humans. *Nature*. 536:285-291.

34. Lal D, May P, Samocha K, Kosmicki J, Robinson EB, Moller R, et al. (2017): Gene family information facilitates variant interpretation and identification of disease-associated genes. *bioRxiv*.

35. Singh T, Walters JTR, Johnstone M, Curtis D, Suvisaari J, Torniainen M, et al. (2017): The contribution of rare variants to risk of schizophrenia in individuals with and without intellectual disability. *Nat Genet*. 49:1167-1173.

36. Samocha KE, Robinson EB, Sanders SJ, Stevens C, Sabo A, McGrath LM, et al. (2014): A framework for the interpretation of de novo mutation in human disease. *Nat Genet*. 46:944-950.

37. Ware JS, Samocha KE, Homsy J, Daly MJ (2015): Interpreting de novo Variation in Human Disease Using denovolyzeR. *Curr Protoc Hum Genet*. 87:7.25.21–27.2515.

38. The Deciphering Developmental Disorders S (2015): Large-scale discovery of novel genetic causes of developmental disorders. *Nature*. 519:223-228.

39. Veltman JA, Brunner HG (2012): De novo mutations in human genetic disease. *Nat Rev Genet*. 13:565-575.

40. Rees E, Kirov G, Walters JT, Richards AL, Howrigan D, Kavanagh DH, et al. (2015): Analysis of exome sequence in 604 trios for recessive genotypes in schizophrenia. *Transl Psychiatry*. 5:e607.

41. Purcell S, Cherny SS, Sham PC (2003): Genetic Power Calculator: design of linkage and association genetic mapping studies of complex traits. *Bioinformatics*. 19:149-150.
